# Supplementary material for: Watching a double strand break repair polymerase insert a pro-mutagenic oxidized nucleotide
Source: Nat Commun. 2021 Apr 6;12:2059. doi: 10.1038/s41467-021-21354-6 (PMC8024293; doi:10.1038/s41467-021-21354-6)
Supplement: Supplementary file 1 — Supplementary Information [file 41467_2021_21354_MOESM1_ESM.pdf]

SUPPLEMENTARY INFORMATION

Watching a Double Strand Break Repair Polymerase Insert a Pro-Mutagenic Oxidized Nucleotide

Jamsen *et al*.

This file includes:

Supplementary Figures 1-7
Supplementary Tables 1-8

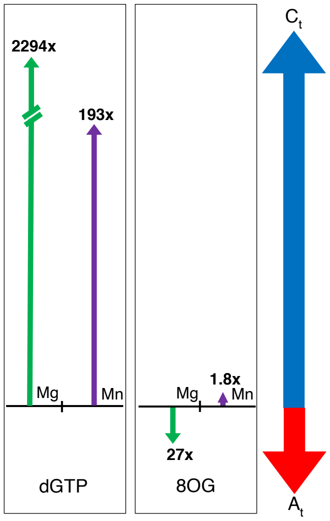

Supplementary Figure 1. dGTP is preferentially inserted opposite C_t in the presence of both Mg^{2+} and Mn^{2+}, whereas 8-oxodGTP is preferentially inserted opposite A_t with Mg^{2+} and C_t with Mn^{2+}. The length of each arrow represents the fold preference for insertion opposite the base indicated by the blue (C_t) and red (A_t) arrows in the presence of 10 mM Mg^{2+} (green arrows) or 1 mM Mn^{2+} (magenta arrows).

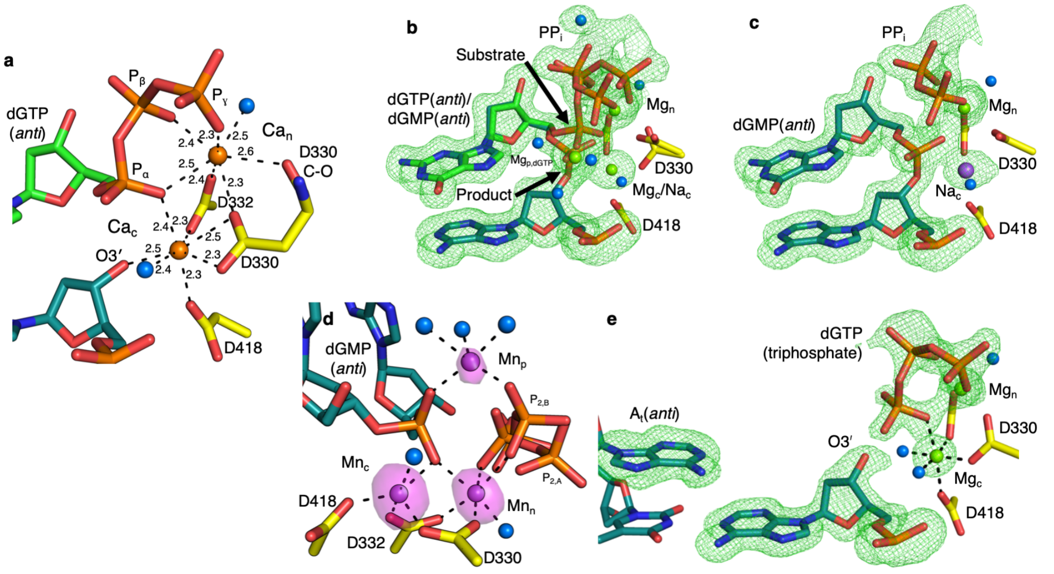

Supplementary Figure 2. Undamaged dGTP insertion. a, Metal coordination in the Ca^{2+}-bound ground state dGTP(*anti*):C_t ternary complex (PDB id 7KSS). Coordination is indicated with black dashes and distances (Å) are labeled. Side-chains are shown in yellow stick representation, DNA is shown in cyan, dGTP is shown in green. **b**, Mg^{2+}-reaction state of dGTP(*anti*):C_t insertion (PDB id 7KSW), where ~50% dGTP insertion has occurred. PP_i was modeled in a conformation where P_2 (former P_γ of dGTP) has rotated ~90° and is stabilized by Mg_p (as in the equivalent Mn^{2+}-insertion). Continuous F_o-F_c density perpendicular to this conformation is observed, that may represent a possible additional PP_i conformation, but is too weak to model. Asp330 can be modeled in two conformations and either coordinates both Mg_n and Mg_c or has rotated ~90° and exhibits a longer coordination distance to Mg_c/Na_c. The simulated annealing (F_o-F_c) omit density (green mesh) shown is contoured at 3.0σ, carve radius 2.0Å. **c**, Mg^{2+}-ternary product complex of the dGTP(*anti*):C_t insertion (PDB id 7KSX). Full product formation has occurred as shown by the lack of density between the phosphate of incorporated dGMP and PP_i in comparison to panel **b**. PP_i and Asp330 adopt rotated conformations. Additional continuous F_o-F_c density perpendicular to PP_i may represent an additional PP_i conformation that was too weak to model. Mg_n is bound to the nucleotide metal site, while Na_c occupies the catalytic site. The simulated annealing (F_o-F_c) omit density (green mesh) shown is contoured at 3.0σ, carve radius 3.0 Å. **d**, Active site anomalous density (magenta surface) for the 8-oxo-dGTP(*anti*):C_t product ternary complex (PDB id 7KSU) contoured at 5.0σ. **e**, Mg^{2+}-ground state dGTP:A_t(*anti*) ternary complex (PDB id 7KT0) after a 60 min soak in a Mg^{2+}-containing cryo-solution. The dGTP triphosphate is fully bound, but F_o-F_c density for the base and sugar are absent. Although Mg_n and Mg_c have exchanged for Ca^{2+}, bond formation is not observed. An additional water molecule coordinates Mg_c. The simulated annealing (F_o-F_c) omit density (green mesh) shown is contoured at 3.0σ, carve radius 2.0 Å.

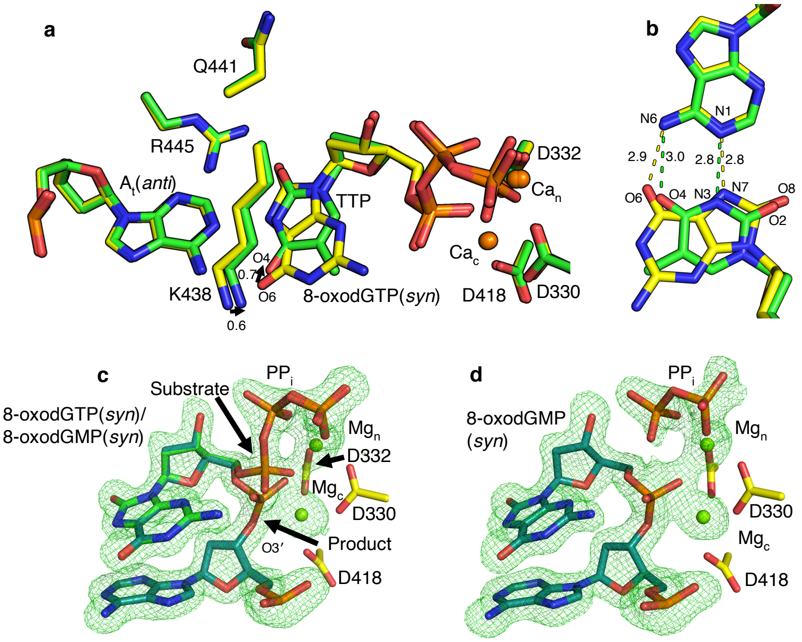

Supplementary Figure 3. 8-oxodGTP(*syn*):A_t insertion. a, 8-oxodGTP(*syn*) mimics TTP(*anti*) in the polymerase active site. Overlay of Ca^{2+}-bound 8-oxodGTP(*syn*):A_t(*anti*) (yellow, PDB id 7KT3) and TTP(*anti*):A_t(*anti*) (green, PDB id 5TXX) ground state ternary complexes. K438 adjusts its position to stabilize the major groove exocyclic oxygen of the incoming dNTP (O6 of 8-oxodGTP(*syn*), O4 of TTP). **b**, Hydrogen bonding through the 8-oxoG(*syn*) Hoogsteen edge is similar to Watson-Crick hydrogen bonding of TTP(*anti*) opposite A_t(*anti*). As shown here, hydrogen bonding of 8-oxoG(*syn*) (yellow dashes) and TTP(*anti*) (green dashes) with N1 and N6 of A_t is similar. Atoms involved in base-pairing are labeled and distances (Å) are indicated. **c**, Mg^{2+}-reaction state of the 8-oxodGTP(*syn*):A_t(*anti*) insertion (PDB id 7KT7) after a 60 min soak in a cryo-solution containing 50 mM Mg^{2+}. Approximately 50% insertion has occurred. **d**, Mg^{2+}-product state of the 8-oxodGTP(*syn*):A_t(*anti*) insertion (PDB id 7KT8). PP_i has partially dissociated. In **c** and **d**, Mg^{2+} is shown as green spheres, side-chains are in yellow stick representation, DNA in cyan, 8-oxo-dGTP in green. The simulated annealing (F_o-F_c) omit density (green mesh) shown is contoured at 3.0σ, carve radius 2.0 Å.

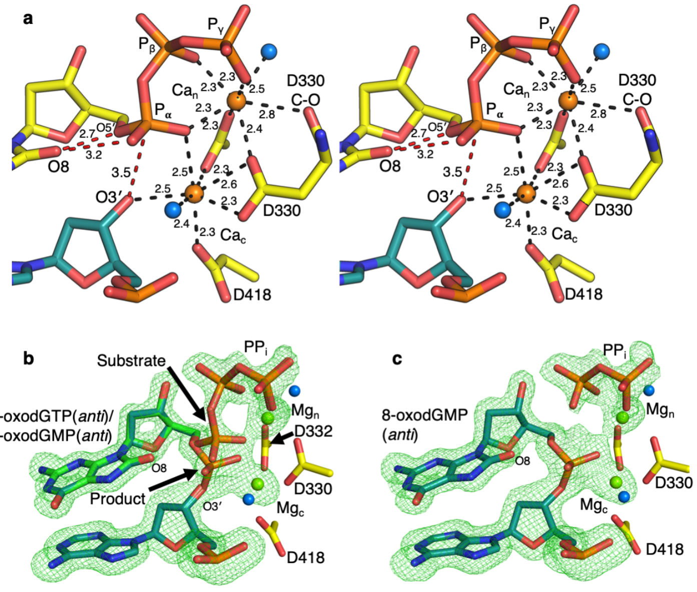

Supplementary Figure 4. 8-oxodGTP(*anti*):C_t insertion. a, Stereo view of metal coordination in the Ca^{2+}:8-oxodGTP(*anti*):C_t(*anti*) ground state ternary complex (PDB id 7KTA). Coordination is shown with black dashes, key distances are shown as red dashes, and distances (Å) are labeled. Protein side-chains are in yellow, DNA is in cyan. Waters are shown as blue spheres. **b**, Mg^{2+}:8-oxodGTP(*anti*):C_t(*anti*) reaction state ternary complex (PDB id 7KTE) displaying ~50% insertion after a 90 min soak in a 50 mM $MgCl_2$ cryo-solution. **c**, Mg^{2+}:8-oxodGTP(*anti*):C_t(*anti*) product state ternary complex (PDB id 7KTF). The Ca^{2+}-ground state ternary complex crystal was soaked in a cryo-solution containing 50 mM $MgCl_2$ for 180 min. In **b** and **c**, Mg^{2+} is shown as green spheres, water molecules as blue spheres. Side-chains are in yellow stick representation, DNA in cyan, 8-oxo-dGTP in green. The simulated annealing (F_o-F_c) omit density (green mesh) shown is contoured at 3.0σ, carve radius 2.0 Å.

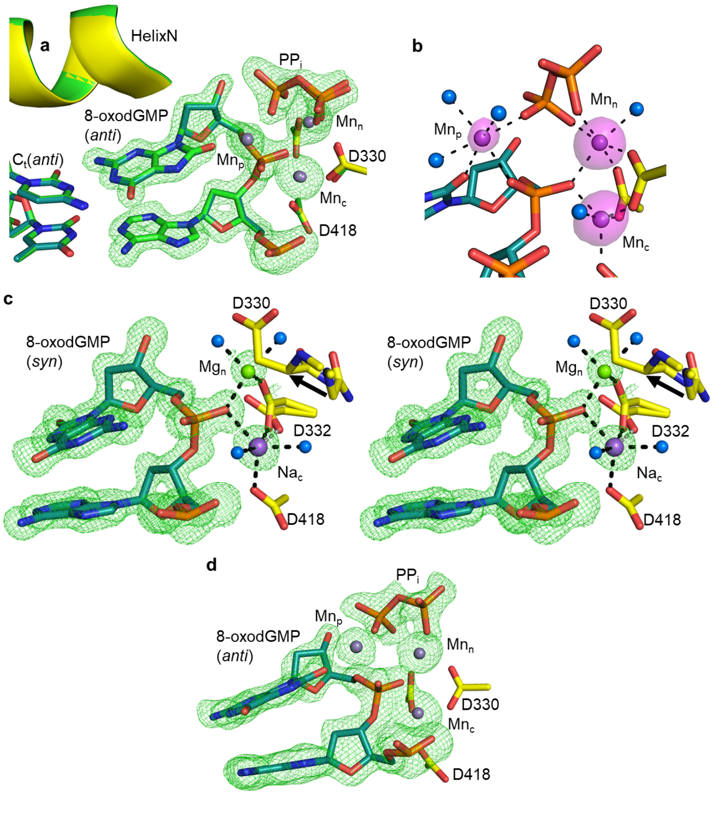

Supplementary Figure 5. Post-catalytic metal and product dynamics. **a**, Product metal modulation of pyrophosphate release. Density for pyrophosphate (PP_i) is absent in all post-catalytic soaks, except the extended (960 min) soak of the Mn^{2+}:8-oxodGTP(*anti*):C_t(*anti*) ternary product complex (PDB id 7KTD; yellow side-chains, cyan DNA). Shown is an overlay with the Mn^{2+}:8-oxodGTP(*anti*):C_t(*anti*) product complex after a 120 min soak (PDB id 7KTC; green side-chains, green DNA). Mn^{2+} atoms are shown as purple spheres. The simulated annealing (F_o-F_c) omit density shown for the primer terminus and active site metals (green mesh) is contoured at 3.0σ, carve radius 2.0 Å. **b**, Anomalous density (magenta surface) at 5.0σ for the extended (960 min) soak of the Mn^{2+}:8-oxodGTP(*anti*):C_t(*anti*) ternary product complex (as in panel **a**). Water molecules are shown as blue spheres. **c**, Stereo view of the 72 hour soak of the Mg^{2+}:8-oxodGTP(*syn*):A_t(*anti*) ternary product complex (PDB id 7KTN). Density for Mg_n (green sphere) at reduced occupancy in the absence of PP_i is still observed, and Na_c (purple sphere) is still bound. The protein backbone from Gln327 to Asp332 is disordered. Metal coordination is shown with black dashes, and with a grey dash for an alternate conformation of Asp332. Water molecules are shown as blue spheres. **d**, Bond formation and product metal at sub-physiological (20 μM) Mn^{2+} concentration. Ca^{2+}-GS ternary complex crystals were soaked in a cryo-solution containing 20 μM Mn^{2+} for 120 min (PDB id 7KTI). The simulated annealing (F_o-F_c) omit density (green mesh) shown is contoured at 3.0σ, carve radius 2.0 Å.

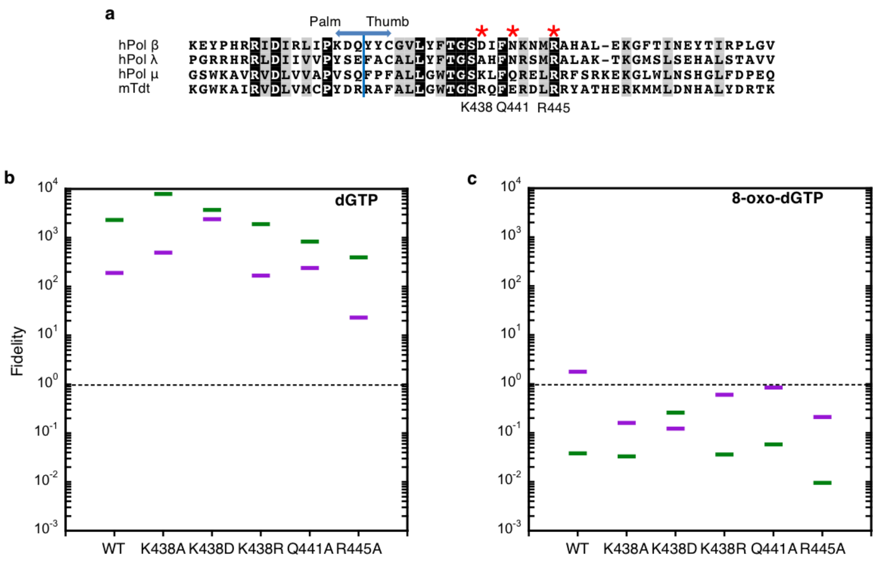

Supplementary Figure 6. Effects of active site substitutions on fidelity of undamaged and oxidized dGTP insertion. a, Multiple sequence alignment of X-family polymerases showing the palm and thumb subdomains. Sequences from representative structures of pols β (PDB id 2FMS), λ (PDB id 1XSN), μ (PDB id 4M04) and Tdt (PDB id 2IYA) were structurally aligned using the palm domains of each polymerase. Red asterisks indicate residues substituted for alanine (K438, Q441, R445), aspartate (K438), or arginine (K438). Fidelity of **b**, dGTP and **c**, 8-oxodGTP insertion in the presence of Mg^{2+} (green lines) or Mn^{2+} (magenta lines) by wild type pol μ and active site variants. Manganese increases fidelity of 8-oxodGTP insertion compared to dGTP insertion for all variants, apart from the K438D variant. Fidelity $((k_{cat}/K_M)_C/(k_{cat}/K_M)_A)$ was calculated by dividing the efficiency of dGTP or 8-oxodGTP insertion opposite C_t by the efficiency for insertion opposite A_t from triplicate measurements.

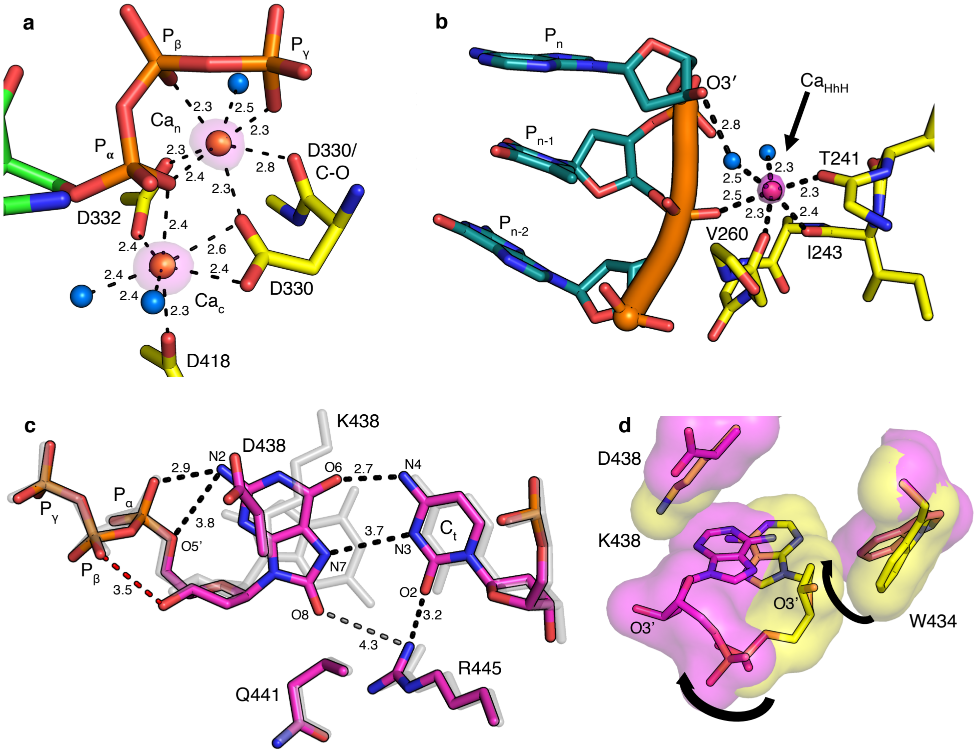

Supplementary Figure 7. Ground state Ca^{2+}:8-oxodGTP(*syn*):C_t(*anti*) ternary complex of the K438D variant (PDB id 7KTJ). a, Metal coordination in the active site. Coordination distances (2.3-2.8 Å) and geometry (7-coordinated) correspond to occupation of the nucleotide (Ca_n) and catalytic metal (Ca_n) sites by Ca^{2+}. Coordination is shown with dashes and distances (Å) are labeled. Anomalous density for Ca^{2+}_{HhH} (orange sphere) is shown contoured at 5.0σ. **b**, Coordination of O3′ by a Ca^{2+} (Ca^{2+}_{HhH}, instead of Na^{+}) atom bound to the Helix-turn-helix motif through a water molecule. Ca^{2+}_{HhH} is coordinated by backbone oxygens of T241, I243 and V260, and two water molecules. Despite the flipped orientation of the primer terminus, base stacking in the primer strand is observed. Hydrogen bonds are shown with dashed lines and distances (Å) are labeled. Anomalous density for Ca^{2+}_{HhH} (orange sphere) is shown contoured at 5.0σ. **c**, Nucleotide interactions in the K438D pre-catalytic ternary complex. Overlay with the wild-type 8-oxodGTP(*anti*):C_t(*anti*) (grey transparent sticks, PDB id 7KTA) Ca^{2+}-ground state displays similar positions of 8-oxodGTP, template base (C_t), Arg445 and Q441. Stabilization of the incoming nucleotide occurs *via* hydrogen bonding through the Hoogsteen edge of 8-oxodGTP, Arg445 and N2 interactions with P_α and O5′. **d**, Overlay of K438D (magenta) and wild-type (yellow) ground state Ca^{2+}:8-oxodGTP:C_t(*anti*) ternary complexes demonstrates a ~180° rotation of the primer terminus. W434 is observed to rotate inward into the space occupied by the primer terminus in the wild-type active site. Side-chains and primer termini are shown in stick representation with atomic volume represented as a surface. Black arrows indicate differences in primer terminal nucleotide and W434.

Supplementary Table 1. Kinetic analysis of dGTP and 8-oxodGTP (8OG) insertion by pol µ.

dNTP	Metal	Template	K_M, µM	k_{cat}, min^{-1}	k_{cat}/K_M, $µM^{-1}min^{-1}$
dGTP	Mn	C_t	0.006 ± 0.001	0.16 ± 0.01	26.9 ± 4.7
		A_t	21.3 ± 2.3	2.98 ± 0.09	0.14 ± 0.02
	Mg	C_t	3.48 ± 0.31	5.70 ± 0.21	1.64 ± 0.16
		A_t	55.6 ± 5.9	0.04 ± 0.01	0.0007 ± 0.0002
8OG	Mn	C_t	5.03 ± 1.19	5.92 ± 0.41	1.18 ± 0.29
		A_t	7.80 ± 2.22	5.14 ± 0.42	0.66 ± 0.20
	Mg	C_t	141 ± 14	1.35 ± 0.04	0.010 ± 0.001
		A_t	31.30 ± 1.53	8.20 ± 0.16	0.26 ± 0.01

Values reported are the mean ± S.E. of three independent measurements.

Supplementary Table 2. Crystallographic statistics.

	dGTP(*anti*):C_t 10 mM Ca^{2+} 20 min	dGTP(*anti*):C_t 10 mM Mn^{2+} 2 min	dGTP(*anti*):C_t 10 mM Mn^{2+} 4 min	dGTP(*anti*):C_t 10 mM Mn^{2+} 960 min	dGTP(*anti*):C_t 10 mM Mg^{2+} 10 min	dGTP(*anti*):C_t 10 mM Mg^{2+} 30 min	dGTP(*anti*):C_t 10 mM Mg^{2+} 960 min
**PDB ID**	7KSS	7KST	7KSU	7KSV	7KSW	7KSX	7KSY
**Data Collection**							
Space Group	$P2_12_12_1$	$P2_12_12_1$	$P2_12_12_1$	$P2_12_12_1$	$P2_12_12_1$	$P2_12_12_1$	$P2_12_12_1$
Cell Dimensions							
a, b, c (Å)	60.023	59.865	59.985	60.002	60.043	60.011	60.316
	68.767	68.831	68.882	68.825	68.736	68.695	62.323
	110.270	110.059	109.747	110.101	110.548	110.525	119.043
α, β, γ (°)	90, 90, 90	90, 90, 90	90, 90, 90	90, 90, 90	90, 90, 90	90, 90, 90	90, 90, 90
Resolution (Å)[1]	50 – 1.50	50 – 1.60	50 – 1.65	50 – 1.64	50 – 1.49	50 – 1.57	50 – 1.58
	(1.56 – 1.50)	(1.66 – 1.60)	(1.71 – 1.65)	(1.70 – 1.64)	(1.54 – 1.49)	(1.63 – 1.57)	(1.63 – 1.58)
R_{sym} or R_{merge}[1]	18.9 (93.7)	7.8 (92.8)	14.3 (97.6)	6.3 (103.2)	9.3 (93.0)	4.6 (84.4)	9.5 (56.9)
$I/\sigma I$[1]	10.9 (2.0)	18.5 (2.1)	11.4 (2.2)	26.3 (1.9)	19.4 (2.4)	31.6 (2.1)	18.6 (3.6)
Completeness (%)[1]	97.8 (97.4)	98.3 (98.3)	99.4 (99.5)	99.8 (99.6)	99.8 (100.0)	99.7 (99.5)	99.9 (100.0)
Redundancy[1]	7.0 (5.1)	5.8 (5.4)	6.8 (6.2)	5.7 (5.2)	7.5 (7.6)	5.3 (5.0)	6.4 (4.7)
No. Unique Refl.[1]	73,209	60,929	55,247	56,860	75,533	64,475	62,460
**Refinement**							
RS : PS (%)	100 : 0	60 : 40	0 : 100	0 : 100	40 : 60	0 : 100	0 : 100
A site occ (%)	100 Ca^{2+}	100 Mn^{2+}	100 Mn^{2+}	100 Mn^{2+}	100 Mg^{2+}	100 Mg^{2+}	100 Mg^{2+}
B site occ (%)	100 Ca^{2+}	100 Mn^{2+}	100 Mn^{2+}	100 Mn^{2+}	100 Mg^{2+}	100 Na^{+}	100 Na^{+}
C site occ (%)	0	40 Mn^{2+}	70 Mn^{2+}	0	60 Mg^{2+}	0	0
Resolution (Å)	42 – 1.50	42 – 1.60	42 – 1.65	35 – 1.64	35 – 1.49	42 – 1.57	35 – 1.58
No. Reflections	71,515	59,855	54,837	56,696	75,335	64,194	62,313
R_{work} / R_{free}	0.16 / 0.18	0.16 / 0.19	0.17 / 0.19	0.16 / 0.19	0.15 / 0.17	0.16 / 0.19	0.16 / 0.17
No. atoms							
Protein / DNA	2683 / 383	2684 / 385	2664 / 364	2628 / 364	2743 / 426	2658 / 405	2632 / 364
dNTP / PP_i / Metal	31 / 0 / 3	31 / 18 / 7	0 / 18 / 8	0 / 0 / 7	31 / 9 / 4	0 / 9 / 3	0 / 0 / 3
Water / Ligands	390 / 63	377 / 73	301 / 51	343 / 41	428 / 69	358 / 31	336 / 33
B-factors							
Protein / DNA	24.8 / 23.9	24.5 / 23.4	31.7 / 30.9	27.0 / 26.5	24.9 / 23.2	27.9 / 26.2	29.0 / 23.1
Me_A / Lig_A[2]	14.9 / 19.3	16.3 / 20.6	20.2 / 22.7	16.3 / 15.4	15.4 / 18.7	18.6 / 21.1	21.9 / 22.7
Me_B / Lig_B[2]	14.4 / 16.2	18.5 / 14.6	19.4 / 21.0	14.1 / 14.2	14.6 / 16.6	17.3 / 19.7	20.5 / 22.6
Me_C / Lig_C[2]	– / –	27.2 / 18.9	32.2 / 31.6	– / –	29.8 / 24.2	– / –	– / –
Water / Ligands[3]	37.1 / 30.2	35.3 / 26.6	39.4 / 32.9	36.0 / 36.8	36.2 / 25.9	38.1 / 32.2	38.8 / 40.2
Wilson B	19.9	20.2	26.2	22.1	20.6	21.9	22.2
R.M.S Deviations							
Bond Lengths (Å)	0.009	0.011	0.009	0.007	0.013	0.014	0.008
Bond Angles (°)	1.083	1.220	0.953	0.951	1.312	1.273	1.067

[1]Data in the highest resolution shell is shown in parentheses.
[2]B-factors for the catalytic metal (Me_A), nucleotide metal (Me_B), product metal (Me_C), and surrounding ligands (Lig_A, Lig_B and Lig_C).
[3]Overall B-factor for ligands/solutes.

Supplementary Table 3. Crystallographic statistics.

	dGTP(*anti*):A_t 10 mM Ca^{2+} 960 min	dGTP(*anti*):A_t 50 mM Mg^{2+} 60 min	dGTP(*anti*):A_t 50 mM Mn^{2+} 180 min	dGTP(*anti*):A_t 50 mM Mn^{2+} 225 min	8OG(*anti*):A_t 20 mM Ca^{2+} 120 min	8OG(*anti*):A_t 10 mM Mn^{2+} 30 min
**PDB ID**	7KSZ	7KT0	7KT1	7KT2	7KT3	7KT4
**Data Collection**						
Space Group	$P2_12_12_1$	$P2_12_12_1$	$P2_12_12_1$	$P2_12_12_1$	$P2_12_12_1$	$P2_12_12_1$
Cell Dimensions						
a, b, c (Å)	59.753	59.918	59.754	59.893	59.804	60.119
	68.778	68.568	68.793	68.866	68.579	68.819
	110.133	110.222	110.223	110.319	110.149	110.296
α, β, γ (°)	90, 90, 90	90, 90, 90	90, 90, 90	90, 90, 90	90, 90, 90	90, 90, 90
Resolution (Å)[1]	50 – 1.42	50 – 1.36	50 – 1.67	50 – 1.50	50 – 1.88	50 – 1.92
	(1.47 – 1.42)	(1.41 – 1.36)	(1.72 – 1.67)	(1.56 – 1.50)	(1.95 – 1.88)	(1.99 – 1.92)
R_{sym} or R_{merge}[1]	9.7 (91.9)	8.9 (78.8)	9.3 (89.5)	5.7 (80.5)	5.9 (39.2)	7.2 (69.9)
$I/\sigma I$[1]	15.1 (1.9)	17.5 (2.1)	18.2 (3.2)	28.1 (2.0)	39.2 (6.0)	24.8 (2.2)
Completeness (%)[1]	99.8 (100.0)	99.8 (98.1)	99.8 (99.3)	99.7 (97.0)	96.1 (90.1)	99.8 (99.7)
Redundancy[1]	5.5 (5.1)	6.9 (4.6)	7.1 (5.7)	6.2 (5.5)	12.5 (8.2)	8.2 (5.1)
No. Unique Refl.[1]	86,689	97,986	53,923	73,871	37,710	35,557
**Refinement**						
RS : PS (%)	100 : 0	100 : 0	40 : 60	0 : 100	100 : 0	40 : 60
A site occ (%)	100 Ca^{2+}	90 Mg^{2+}	100 Mn^{2+}	100 Mn^{2+}	100 Ca^{2+}	100 Mn^{2+}
B site occ (%)	100 Ca^{2+}	70 Mg^{2+}	100 Mn^{2+}	90 Mn^{2+}	100 Ca^{2+}	100 Mn^{2+}
C site occ (%)	0	0	0	0	0	0
Resolution (Å)	33 – 1.42	34 – 1.36	43 – 1.67	35 – 1.50	25 – 1.88	21 – 1.92
No. Reflections	86,453	97,642	53,737	73,590	36,063	35,285
R_{work} / R_{free}	0.16 / 0.18	0.16 / 0.18	0.17 / 0.19	0.17 / 0.19	0.16 / 0.19	0.17 / 0.20
No. atoms						
Protein / DNA	2691 / 385	2787 / 344	2675 / 428	2616 / 407	2722 / 401	2622 / 429
dNTP / PP_i / Metal	31 / 0 / 3	31 / 0 / 3	31 / 0 / 7	0 / 0 / 8	32 / 0 / 4	32 / 9 / 5
Water / Ligands	444 / 63	431 / 44	297 / 97	345 / 57	375 / 68	335 / 62
B-factors						
Protein / DNA	19.9 / 19.9	23.7 / 22.1	31.0 / 31.7	26.1 / 27.3	22.2 / 21.2	25.6 / 25.1
Me_A / Lig_A[2]	12.3 / 17.6	20.0 / 24.6	20.2 / 21.2	15.7 / 19.7	12.6 / 14.7	20.4 / 15.5
Me_B / Lig_B[2]	11.6 / 14.4	18.6 / 22.0	18.7 / 19.8	12.7 / 16.5	12.8 / 14.4	16.3 / 17.0
Me_C / Lig_C[2]	– / –	– / –	– / –	– / –	– / –	– / –
Water / Ligands[3]	32.5 / 32.3	34.8 / 32.4	40.4 / 32.0	35.4 / 29.4	30.3 / 25.1	32.1 / 24.7
Wilson B	16.8	18.7	24.6	20.6	21.0	23.5
R.M.S Deviations						
Bond Lengths (Å)	0.010	0.008	0.014	0.010	0.014	0.009
Bond Angles (°)	1.197	1.038	1.293	1.107	1.114	0.733

[1]Data in the highest resolution shell is shown in parentheses.
[2]B-factors for the catalytic metal (Me_A), nucleotide metal (Me_B), product metal (Me_C), and surrounding ligands (Lig_A, Lig_B and Lig_C).
[3]Overall B-factor for ligands/solutes.

Supplementary Table 4. Crystallographic statistics.

	8OG(*anti*):A_t 10 mM Mn^{2+} 120 min	8OG(*anti*):A_t 10 mM Mn^{2+} 960 min	8OG(*anti*):A_t 50 mM Mg^{2+} 60 min	8OG(*anti*):A_t 50 mM Mg^{2+} 180 min	8OG(*anti*):A_t 50 mM Mg^{2+} 960 min	8OG(*anti*):C_t 20 mM Ca^{2+} 120 min
**PDB ID**	7KT5	7KT6	7KT7	7KT8	7KT9	7KTA
**Data Collection**						
Space Group	$P2_12_12_1$	$P2_12_12_1$	$P2_12_12_1$	$P2_12_12_1$	$P2_12_12_1$	$P2_12_12_1$
Cell Dimensions						
a, b, c (Å)	59.996	60.081	59.908	59.891	60.283	59.905
	68.701	68.692	68.531	68.503	68.316	68.718
	110.125	110.490	110.589	110.793	111.407	110.398
α, β, γ (°)	90, 90, 90	90, 90, 90	90, 90, 90	90, 90, 90	90, 90, 90	90, 90, 90
Resolution (Å)[1]	50 – 1.46	50 – 1.87	50 – 1.76	50 – 1.70	50 – 1.48	50 – 1.84
	(1.51 – 1.46)	(1.94 – 1.87)	(1.82 – 1.76)	(1.76 – 1.70)	(1.54 – 1.48)	(1.91 – 1.84)
R_{sym} or R_{merge}[1]	7.0 (76.3)	7.6 (84.1)	11.0 (93.5)	5.6 (77.2)	12.5 (57.1)	8.3 (93.5)
$I/\sigma I$[1]	17.6 (2.1)	31.9 (2.1)	15.6 (2.1)	22.4 (2.0)	11.9 (3.2)	23.7 (2.1)
Completeness (%)[1]	99.0 (97.5)	99.9 (100.0)	99.7 (99.9)	97.1 (97.3)	99.7 (99.9)	100.0 (99.9)
Redundancy[1]	5.2 (4.6)	11.5 (6.8)	5.1 (4.7)	4.8 (4.0)	5.8 (5.9)	7.2 (6.3)
No. Unique Refl.[1]	79,653	38,602	45,847	50,761	77,089	40,062
**Refinement**						
RS : PS (%)	0 : 100	0 : 100	50 : 50	0 : 100	0 : 100	100 : 0
A site occ (%)	90 Mn^{2+}	90 Mn^{2+}	100 Mg^{2+}	100 Mg^{2+}	100 Na^{+}	100 Ca^{2+}
B site occ (%)	100 Mn^{2+}	100 Mn^{2+}	100 Mg^{2+}	100 Mg^{2+}	50 Mg^{2+}	100 Ca^{2+}
C site occ (%)	0	0	0	0	0	0
Resolution (Å)	35 – 1.46	21 – 1.87	35 – 1.76	42 – 1.70	45 – 1.48	32 – 1.84
No. Reflections	78,790	38,376	45,654	49,210	76,759	39,972
R_{work} / R_{free}	0.16 / 0.19	0.17 / 0.19	0.16 / 0.19	0.17 / 0.19	0.16 / 0.18	0.17 / 0.20
No. atoms						
Protein / DNA	2652 / 408	2676 / 408	2681 / 429	2609 / 408	2788 / 408	2661 / 383
dNTP / PP_i / Metal	0 / 0 / 8	0 / 0 / 7	32 / 9 / 3	0 / 9 / 3	0 / 0 / 3	32 / 0 / 3
Water / Ligands	377 / 40	313 / 32	359 / 71	293 / 32	465 / 25	190 / 51
B-factors						
Protein / DNA	24.9 / 24.3	30.3 / 30.5	24.7 / 21.2	31.1 / 27.8	21.2 / 19.8	38.6 / 35.8
Me_A / Lig_A[2]	14.9 / 17.3	18.6 / 19.2	13.1 / 20.3	23.0 / 33.9	15.5 / 16.6	27.2 / 35.1
Me_B / Lig_B[2]	13.8 / 14.6	18.3 / 18.2	20.4 / 17.7	26.9 / 27.1	18.5 / 20.3	28.0 / 28.2
Me_C / Lig_C[2]	– / –	– / –	– / –	– / –	– / –	– / –
Water / Ligands[3]	35.4 / 31.4	38.2 / 35.3	35.7 / 23.0	38.4 / 34.1	33.4 / 30.1	44.3 / 39.4
Wilson B	19.4	25.7	20.8	25.2	16.6	33.7
R.M.S Deviations						
Bond Lengths (Å)	0.010	0.015	0.007	0.009	0.013	0.008
Bond Angles (°)	1.256	1.072	1.073	1.281	1.335	1.028

[1]Data in the highest resolution shell is shown in parentheses.
[2]B-factors for the catalytic metal (Me_A), nucleotide metal (Me_B), product metal (Me_C), and surrounding ligands (Lig_A, Lig_B and Lig_C).
[3]Overall B-factor for ligands/solutes.

Supplementary Table 5. Crystallographic statistics.

	8OG(*anti*):C_t 10 mM Mn^{2+} 40 min	8OG(*anti*):C_t 10 mM Mn^{2+} 120 min	8OG(*anti*):C_t 10 mM Mn^{2+} 960 min	8OG(*anti*):C_t 50 mM Mg^{2+} 90 min	8OG(*anti*):C_t 50 mM Mg^{2+} 180 min	8OG(*anti*):C_t 50 mM Mg^{2+} 960 min
**PDB ID**	7KTB	7KTC	7KTD	7KTE	7KTF	7KTG
**Data Collection**						
Space Group	$P2_12_12_1$	$P2_12_12_1$	$P2_12_12_1$	$P2_12_12_1$	$P2_12_12_1$	$P2_12_12_1$
Cell Dimensions						
a, b, c (Å)	60.278	60.272	60.316	59.940	59.947	60.070
	62.319	62.354	62.420	68.523	68.515	68.523
	119.130	118.901	119.135	110.605	110.696	110.738
α, β, γ (°)	90, 90, 90	90, 90, 90	90, 90, 90	90, 90, 90	90, 90, 90	90, 90, 90
Resolution (Å)[1]	50 – 1.58	50 – 1.65	50 – 1.55	50 – 1.48	50 – 1.49	50 – 1.45
	(1.63 – 1.58)	(1.71 – 1.65)	(1.61 – 1.55)	(1.53 – 1.48)	(1.54 – 1.49)	(1.50 – 1.45)
R_{sym} or R_{merge}[1]	5.6 (98.2)	9.8 (70.9)	7.8 (63.4)	13.6 (88.6)	8.1 (95.9)	12.7 (72.1)
$I/\sigma I$[1]	31.0 (2.1)	16.2 (2.1)	22.7 (2.6)	12.9 (2.4)	20.3 (1.8)	11.8 (2.2)
Completeness (%)[1]	100.0 (100.0)	99.9 (99.8)	99.8 (99.1)	99.9 (99.9)	97.4 (95.9)	99.0 (97.3)
Redundancy[1]	5.9 (5.6)	5.9 (4.4)	7.1 (5.4)	6.2 (5.9)	5.9 (4.8)	5.6 (4.2)
No. Unique Refl.[1]	62,629	54,443	65,881	76,763	75,307	82,346
**Refinement**						
RS : PS (%)	40 : 60	0 : 100	0 : 100	40 : 60	0 : 100	0 : 100
A site occ (%)	100 Mn^{2+}	100 Mn^{2+}	90 Mn^{2+}	100 Mg^{2+}	100 Mg^{2+}	100 Mg^{2+}
B site occ (%)	100 Mn^{2+}	100 Mn^{2+}	100 Mn^{2+}	100 Mg^{2+}	100 Mg^{2+}	80 Mg^{2+}
C site occ (%)	60 Mn^{2+}	40 Mn^{2+}	50 Mn^{2+}	0	0	0
Resolution (Å)	35 – 1.58	35 – 1.65	43 – 1.55	35 – 1.48	35 – 1.49	43 – 1.45
No. Reflections	62,530	52,021	65,671	76,617	73,280	81,444
R_{work} / R_{free}	0.16 / 0.18	0.16 / 0.18	0.16 / 0.18	0.16 / 0.18	0.17 / 0.19	0.16 / 0.18
No. atoms						
Protein / DNA	2693 / 424	2650 / 403	2611 / 403	2783 / 427	2689 / 365	2788 / 406
dNTP / PP_i / Metal	32 / 9 / 10	0 / 9 / 8	0 / 9 / 7	32 / 9 / 3	0 / 9 / 3	0 / 0 / 3
Water / Ligands	382 / 76	415 / 49	348 / 38	460 / 81	423 / 54	440 / 36
B-factors						
Protein / DNA	27.9 / 25.0	16.7 / 13.2	29.3 / 25.6	22.2 / 21.0	22.9 / 20.7	23.5 / 22.0
Me_A / Lig_A[2]	21.1 / 21.3	9.2 / 11.5	21.4 / 26.0	20.1 / 25.4	22.7 / 30.1	18.5 / 24.9
Me_B / Lig_B[2]	17.7 / 18.4	6.7 / 9.0	18.7 / 21.8	23.4 / 22.7	27.5 / 24.8	17.0 / 27.5
Me_C / Lig_C[2]	27.1 / 23.3	9.5 / 15.3	25.3 / 24.3	– / –	– / –	– / –
Water / Ligands[3]	39.7 / 34.0	29.9 / 28.7	39.7 / 40.7	34.6 / 31.0	34.5 / 32.9	35.4 / 32.9
Wilson B	20.6	12.5	23.2	18.1	19.3	18.3
R.M.S Deviations						
Bond Lengths (Å)	0.011	0.013	0.009	0.013	0.012	0.012
Bond Angles (°)	1.233	1.355	1.138	1.343	1.274	1.312

[1]Data in the highest resolution shell is shown in parentheses.
[2]B-factors for the catalytic metal (Me_A), nucleotide metal (Me_B), product metal (Me_C), and surrounding ligands (Lig_A, Lig_B and Lig_C).
[3]Overall B-factor for ligands/solutes.

Supplementary Table 6. Crystallographic statistics.

	8OG(*anti*):C_t<br>10 mM Mg^{2+}<br>2160 min	8OG(*anti*):C_t<br>20 μM Mn^{2+}<br>120 min	K438D<br>8OG(*anti*):C_t<br>20 mM Ca^{2+}<br>120 min	K438D<br>8OG(*anti*):C_t<br>50 mM Mn^{2+}<br>30 min	K438D<br>8OG(*anti*):C_t<br>50 mM Mn^{2+}<br>90 min	K438D<br>8OG(*syn*):C_t<br>50 mM Mg^{2+}<br>90 min	8OG(*anti*):A_t<br>10 mM Mg^{2+}<br>2160 min
**PDB ID**	7KTH	7KTI	7KTJ	7KTM	7KTL	7KTK	7KTN
**Data Collection**							
Space Group	$P2_12_12_1$	$P2_12_12_1$	$P2_12_12_1$	$P2_12_12_1$	$P2_12_12_1$	$P2_12_12_1$	$P2_12_12_1$
Cell Dimensions							
a, b, c (Å)	60.089	59.965	60.020	60.241	60.271	60.109	60.229
	68.373	68.716	62.099	62.223	62.099	61.775	68.293
	111.095	110.586	118.212	118.797	118.774	118.076	111.412
α, β, γ (°)	90, 90, 90	90, 90, 90	90, 90, 90	90, 90, 90	90, 90, 90	90, 90, 90	90, 90, 90
Resolution (Å)[1]	50 – 1.48	50 – 1.57	50 – 1.45	50 – 1.53	50 – 1.42	50 – 1.42	50 – 1.33
	(1.53 – 1.48)	(1.63 – 1.57)	(1.50 – 1.45)	(1.58 – 1.53)	(1.47 – 1.42)	(1.47 – 1.42)	(1.38 – 1.33)
R_{sym} or R_{merge}[1]	8.6 (91.1)	5.8 (88.3)	6.6 (85.4)	8.5 (94.9)	6.9 (88.4)	8.9 (95.3)	10.3 (49.7)
$I/\sigma I$[1]	17.0 (2.3)	27.7 (2.1)	23.2 (2.1)	17.8 (2.6)	22.6 (2.1)	19.7 (2.2)	14.0 (2.6)
Completeness (%)[1]	99.7 (100.0)	99.9 (100.0)	91.6 (92.3)	99.2 (99.0)	99.5 (100.0)	99.9 (99.9)	98.9 (88.4)
Redundancy[1]	5.7 (5.5)	6.3 (6.2)	6.7 (6.7)	6.2 (6.4)	6.3 (6.6)	6.1 (5.4)	5.6 (3.9)
No. Unique Refl.[1]	77,588	64,503	78,612	68,341	84,712	83,863	106,016
**Refinement**							
RS : PS (%)	0 : 100	0 : 100	100 : 0	40 : 60	0 : 100	100 : 0	0 : 100
A site occ (%)	100 Na^+	70 Mn^{2+}	100 Ca^{2+}	100 Mn^{2+}	100 Mn^{2+}	100 Mg^{2+}	100 Na^+
B site occ (%)	60 Mg^{2+}	90 Mn^{2+}	100 Ca^{2+}	100 Mn^{2+}	100 Mn^{2+}	100 Mg^{2+}	40 Mg^{2+}
C site occ (%)	0	40 Mn^{2+}	0	60 Mn^{2+}	70 Mn^{2+}	0	0
Resolution (Å)	42 – 1.48	35 – 1.57	35 – 1.45	35 – 1.53	35 – 1.42	33 – 1.42	35 – 1.33
No. Reflections	77,291	64,388	71,980	67,709	84,219	83,705	104,796
R_{work} / R_{free}	0.17 / 0.19	0.16 / 0.18	0.16 / 0.18	0.16 / 0.18	0.16 / 0.17	0.14 / 0.17	0.14 / 0.16
No. atoms							
Protein / DNA	2783 / 406	2701 / 406	2732 / 342	2665 / 444	2673 / 403	2787 / 342	2845 / 367
dNTP / PP_i / Metal	0 / 0 / 3	0 / 9 / 4	32 / 0 / 4	32 / 9 / 10	0 / 9 / 10	32 / 0 / 3	0 / 0 / 3
Water / Ligands	457 / 30	412 / 41	397 / 68	391 / 70	407 / 39	332 / 83	485 / 21
B-factors							
Protein / DNA	22.3 / 20.7	25.5 / 24.8	24.5 / 20.7	26.2 / 23.1	25.1 / 21.7	23.2 / 21.1	20.3 / 18.9
Me_A / Lig_A[2]	22.3 / 23.8	19.6 / 29.5	15.9 / 21.5	17.4 / 17.6	17.0 / 17.5	16.0 / 18.6	15.0 / 15.7
Me_B / Lig_B[2]	26.5 / 37.5	20.6 / 24.1	16.0 / 17.6	15.8 / 16.9	14.6 / 16.3	15.7 / 16.8	12.9 / 15.3
Me_C / Lig_C[2]	– / –	21.7 / 31.3	– / –	19.1 / 20.2	15.5 / 17.8	– / –	– / –
Water / Ligands[3]	33.8 / 30.3	36.6 / 35.2	35.0 / 32.4	38.2 / 26.7	36.5 / 30.5	35.8 / 39.2	32.7 / 25.9
Wilson B	18.6	19.9	18.8	20.2	19.2	18.6	15.7
R.M.S Deviations							
Bond Lengths (Å)	0.014	0.008	0.011	0.013	0.012	0.009	0.012
Bond Angles (°)	1.384	1.097	1.227	1.543	1.387	1.212	1.254

[1]Data in the highest resolution shell is shown in parentheses.
[2]B-factors for the catalytic metal (Me_A), nucleotide metal (Me_B), product metal (Me_C), and surrounding ligands (Lig_A, Lig_B and Lig_C).
[3]Overall B-factor for ligands/solutes.

Supplementary Table 7. Effects of active site substitutions on kinetic parameters for dGTP insertion by pol μ.

Mutant	Metal	Template	K_M, μM	k_{cat}, min^{-1}	k_{cat}/K_M, μM^{-1}min^{-1}
WT	Mn	C_t	0.006 ± 0.001	0.16 ± 0.01	26.9 ± 4.7
		A_t	21.3 ± 2.3	2.98 ± 0.09	0.14 ± 0.02
	Mg	C_t	3.48 ± 0.31	5.70 ± 0.21	1.64 ± 0.16
		A_t	55.6 ± 5.9	0.04 ± 0.01	0.0007 ± 0.0002
K438A	Mn	C_t	0.026 ± 0.003	0.30 ± 0.01	11.54 ± 1.39
		A_t	56.8 ± 3.7	1.33 ± 0.03	0.023 ± 0.002
	Mg	C_t	5.13 ± 0.21	6.92 ± 0.08	1.35 ± 0.06
		A_t	200 ± 40	0.033 ± 0.002	0.00017 ± 0.00003
K438D	Mn	C_t	0.34 ± 0.04	3.28 ± 0.08	9.65 ± 0.89
		A_t	87.5 ± 7.7	0.35 ± 0.01	0.0040 ± 0.0004
	Mg	C_t	81.6 ± 5.3	5.76 ± 0.16	0.071 ± 0.005
		A_t	160 ± 40	0.003 ± 0.0003	0.000019 ± 0.00005
K438R	Mn	C_t	0.017 ± 0.001	0.19 ± 0.01	11.81 ± 0.88
		A_t	14.2 ± 1.5	0.94 ± 0.03	0.066 ± 0.007
	Mg	C_t	4.94 ± 0.30	6.40 ± 0.12	1.30 ± 0.08
		A_t	39.5 ± 5.9	0.027 ± 0.001	0.00068 ± 0.0001
Q441A	Mn	C_t	0.022 ± 0.002	0.260 ± 0.006	11.82 ± 1.11
		A_t	44.6 ± 5.4	2.18 ± 0.08	0.049 ± 0.006
	Mg	C_t	9.12 ± 0.31	6.22 ± 0.07	0.68 ± 0.02
		A_t	29.6 ± 6.7	0.024 ± 0.001	0.00081 ± 0.00019
R445A	Mn	C_t	0.096 ± 0.012	1.46 ± 0.07	15.21 ± 2.04
		A_t	12.9 ± 1.4	8.44 ± 0.28	0.65 ± 0.07
	Mg	C_t	11.8 ± 1.7	3.36 ± 0.18	0.28 ± 0.04
		A_t	102 ± 10	0.072 ± 0.002	0.0007 ± 0.0001

Values reported are the mean ± S.E. of three independent measurements.

Supplementary Table 8. Effects of active site substitutions on kinetic parameters for 8-oxodGTP insertion by pol μ.

Mutant	Metal	Template	K_M, μM	k_{cat}, min^{-1}	k_{cat}/K_M, $\mu M^{-1}min^{-1}$
WT	Mn	C_t	5.03 ± 1.19	5.92 ± 0.41	1.18 ± 0.29
		A_t	7.80 ± 2.22	5.14 ± 0.42	0.66 ± 0.20
	Mg	C_t	141 ± 14	1.35 ± 0.04	0.010 ± 0.001
		A_t	31.30 ± 1.53	8.20 ± 0.16	0.26 ± 0.01
K438A	Mn	C_t	0.84 ± 0.16	1.30 ± 0.07	1.55 ± 0.31
		A_t	0.086 ± 0.007	0.83 ± 0.02	9.62 ± 0.82
	Mg	C_t	64.8 ± 6.0	0.36 ± 0.01	0.006 ± 0.001
		A_t	23.7 ± 0.8	4.28 ± 0.04	0.18 ± 0.01
K438D	Mn	C_t	19.8 ± 3.2	2.08 ± 0.15	0.11 ± 0.02
		A_t	8.74 ± 0.16	7.82 ± 0.05	0.89 ± 0.02
	Mg	C_t	371 ± 45	0.41 ± 0.01	0.0011 ± 0.0001
		A_t	392 ± 48	16.8 ± 0.9	0.0043 ± 0.0006
K438R	Mn	C_t	6.30 ± 0.22	3.72 ± 0.04	0.59 ± 0.02
		A_t	6.18 ± 2.03	5.98 ± 0.61	0.97 ± 0.33
	Mg	C_t	157 ± 9	0.57 ± 0.01	0.004 ± 0.002
		A_t	54.8 ± 4.1	5.40 ± 0.10	0.099 ± 0.008
Q441A	Mn	C_t	4.88 ± 0.54	7.82 ± 0.25	1.60 ± 0.18
		A_t	4.29 ± 0.90	8.08 ± 0.48	1.88 ± 0.41
	Mg	C_t	120 ± 8	0.64 ± 0.01	0.0053 ± 0.0004
		A_t	64.6 ± 4.5	5.88 ± 0.13	0.091 ± 0.007
R445A	Mn	C_t	5.37 ± 0.16	3.26 ± 0.03	0.63 ± 0.02
		A_t	2.94 ± 0.24	8.76 ± 0.18	2.98 ± 0.25
	Mg	C_t	147 ± 33	0.035 ± 0.003	0.00024 ± 0.00006
		A_t	90 ± 4	2.22 ± 0.03	0.025 ± 0.001

Values reported are the mean ± S.E. of three independent measurements.
